# Neuropathic-like Pain in Fibrous Dysplasia/McCune-Albright Syndrome

**DOI:** 10.1210/clinem/dgac120

**Published:** 2022-03-09

**Authors:** Tiahna L Spencer, Laura Watts, Anushka Soni, Rafael Pinedo-Villanueva, Anne-Marie Heegaard, Alison M Boyce, M Kassim Javaid

**Affiliations:** 1 Skeletal Disorders and Mineral Homeostasis Section, National Institute of Dental and Craniofacial Research, National Institutes of Health, Bethesda, MD, USA; 2 Department of Metabolism, Digestion and Reproduction, Imperial College London, Hammersmith Campus, Du Cane Road, London, UK; 3 Botnar Research Centre, Nuffield Department of Orthopaedics, Rheumatology and Musculoskeletal Sciences, University of Oxford, Oxford, UK; 4 Department of Drug Design and Pharmacology, University of Copenhagen, Copenhagen, Denmark; 5 Metabolic Bone Disorders Unit, National Institute of Dental and Craniofacial Research, National Institutes of Health, Bethesda, MD, USA

**Keywords:** fibrous dysplasia, McCune-Albright syndrome, neuropathic-like pain, nociceptive pain, quality of life, mental health

## Abstract

**Context:**

Pain is a major symptom in adults with fibrous dysplasia/McCune-Albright syndrome (FD/MAS) and response to current treatments, including bisphosphonates and standard analgesics (nonsteroidal anti-inflammatory drugs and opiates) is unpredictable. No studies have explored whether the type of pain is variable in this patient group.

**Objective:**

To determine the frequency of neuropathic-like pain in patients with FD/MAS.

**Design:**

Retrospective, dual registry study.

**Setting:**

Community.

**Patients:**

FD/MAS online registries: the US-based Familial Dysautonomia Foundation (FDF) and the UK-based Rare and Undiagnosed Diseases (RUDY) study.

**Intervention:**

Subjects completed questionnaires to evaluate the presence of features of neuropathic-like pain (painDETECT) and the impact on sleep quality (Pittsburgh Sleep Quality Index) and mental health (Hospital Anxiety and Depression Scale). Descriptive statistics were used to characterize the prevalence and associated burden of neuropathic-like pain.

**Main Outcome Measures:**

Incidence of neuropathic, nociceptive, and unclear pain.

**Results:**

Of 249 participants, one third experienced neuropathic-like pain. This group had statistically significantly (*P* < 0.001) worse mental well-being and sleep in comparison to those with predominately nociceptive pain.

**Conclusions:**

Neuropathic-like pain is common in patients with FD/MAS and associated with worse quality of life. Evaluation of pain in patients with FD/MAS should include assessment of neuropathic-like pain to guide personalized approaches to treatment and inform future research.

Fibrous dysplasia/McCune-Albright Syndrome (FD/MAS) is a rare disease resulting from somatic gain-of-function *GNAS* mutations (1), which contributes to constitutive activation of G-protein coupled receptors. Within bone, this activation prevents differentiation of osteoprogenitors, resulting in the replacement of bone and marrow with expansile fibrotic tissue. These lesions can occur in 1 location (monostotic FD) or affect multiple bones (polyostotic FD) (2). Patients can experience fractures, deformity, pain, and disability due to these lesions. MAS occurs when there is additional extraskeletal involvement, typically skin hyperpigmentation and hyperfunctioning endocrinopathies (3). Prior reports have shown that up to 80% of adults with FD/MAS experience pain (4), and the responses to treatments (bisphosphonates, denosumab, nonsteroidal anti-inflammatory drugs, and opiates) are variable and often suboptimal (5). A randomized control trial of high-dose oral alendronate did not reduce pain (6).

The mechanisms underlying pain in FD have not been clarified. Pain severity does not correlate to skeletal disease burden or bone biomarkers (7-9). The contribution of nociceptive vs neuropathic-like pain has not been determined in FD/MAS, and it is unknown whether differences in pain type might explain the variable response to the standard treatments typically given for nociceptive pain. However, *GNAS* mutation bearing skeletal progenitor cells have been shown to secrete interleukin-6 (IL-6), and it has been shown in other disorders that IL-6 is associated with neuropathic and other pain states (5, 10-12). The aim of this study was therefore to describe the prevalence and associated impact of neuropathic-like pain in FD/MAS on quality of life.

## Materials and Methods

### Data Source

Participant data came from 2 community-based registries: the Fibrous Dysplasia Foundation (FDF) FD/MAS Patient Registry and the UK-based Rare Undiagnosed Diseases Study (RUDY) (13). Both registries are web-based with participants directly entering data. The FDF is accessed by patients through the FDF website (https://www.fdmasregistry.org/) and promoted via newsletters and social media to patients with FD/MAS from the United States and internationally, including the United Kingdom. Participants give online consent for their data to be collected and used for research. RUDY is accessed through its own web address (www.rudystudy.org) and promoted to patients with FD/MAS by the Fibrous Dysplasia Support Society UK, as well as social media. Participants are required to enter the name of their rare disease, and this is linked to the Orphanet ontology (14). The participants then complete online questionnaires, including the painDETECT questionnaire (PD-Q) (15).

The PD-Q is a 12-item questionnaire that has been used to record the intensity of their current pain (ranging from 0-10), as well as average and strongest pain during the past four weeks. In addition, the PD-Q uses questions concerning the quality of pain to discriminate whether a subject is experiencing nociceptive pain or features suggestive of neuropathic-like pain. The PD-Q was developed to assess neuropathic pain in back pain (15, 16) and has also been adapted and validated as a measure of central sensitization in the context of osteoarthritis (17). (18, 19) Using the published scoring algorithm (15), which is based on 3 domains (sensory symptoms, pain course pattern, and pain radiation), participants were divided into 3 distinct groups: scores of 0 to 12 are indicative of nociceptive pain; scores of 13 to 18 are classed as unclear, where a neuropathic component may be present; and scores of 19 to 38 indicate neuropathic-like pain is likely. The scores are dependent on the responses given to the questions, which elicit the type of pain experienced, for example if there is a component of numbness or burning. Participants were excluded from the analyses if they did not complete the PD-Q questionnaire, had missing demographic data (sex or age), or were less than 14 years old. All participants gave informed consent (FDF IRB approval 2018-56-FDF, RUDY LREC 14/SC/0126 & 17/SC/0501).

### Outcome Measures

Hospital Anxiety and Depression Scale (HADS) was used to evaluate the severity of anxiety and depression in the last week (20, 21). It is comprised of 14 questions, 7 to evaluate anxiety and 7 to evaluate depression. Written responses were converted to their numerical correlate according to the survey’s algorithm and sum of the scores, per category, were used to determine the final response. Scores less than 8 are normal, 8 to 10 mild, 11 to 14 moderate, and 15 to 21 severe depression or anxiety.

Pittsburgh Sleep Quality Index (PSQI) was used to evaluate quality of sleep over the previous 4 weeks (22, 23). This 19-item survey evaluates 7 components of sleep: subjective sleep quality, time to sleep, sleep duration, sleep disturbances, use of medications, daytime dysfunction, and subjective fatigue. Using the original scoring methods, a final result of greater than 5 is indicative of poor sleep, but according to more recent studies, greater than 8 is a more accurate threshold for sleep impairment and was used in this study (24).

The Medical Outcomes Study 36-Item Short Form Health Survey was used as a general measure of quality of life (25, 26). It comprises 36 questions that contribute to 8 different scales examining aspects of physical and mental health. Physical functioning, role limitations due to physical health and, emotional problems, energy/fatigue, emotional well-being, pain, and general health were the 7 scales were used in this study. The RUDY study used Short Form 36 (SF-36) version 1, and FDF used SF-36 version 2. As there is no cross-walking algorithm between SF-36 versions, each cohort was scored using their respective published version-specific scoring algorithm (27) to produce scores from 0 to 100. Higher scores indicate better quality of life in the respective dimension, and a score of 50 represents the mean score of the reference population.

### Statistical Analysis

Descriptive statistics included Pearson correlation, *t*-tests, and analysis of variance, used for parametric outcome measures, and Spearman correlations, Mann-Whitney tests, and the Kruskal-Wallis tests were used for nonparametric outcomes. Categorical results were evaluated by use of chi-squared tests (sex, pain type, FD type) and Fisher’s exact test when individual cell counts were <10. Multivariate logistic regression analyses were performed with nociceptive pain as the reference group to identify independent predictors of other aspects of quality of life, adjusting for sex, age, and average strongest pain during the previous four weeks. As a sensitivity analysis, models were repeated in individual cohorts. Correction for multiple testing was completed in the via the GraphPad Prism program. Significance was determined as *P* < 0.05. Analyses were completed on GraphPad Prism 7 (GraphPad Software, San Diego, CA, USA) and Stata Intercooled version 15.1 (College Station, TX, USA).

## Results

### Patient Characteristics

A total of 249 participants were eligible for this study: 181 from the FDF and 68 from RUDY. Women made up 82.3% of the cohort and had a mean age of 41 years (range 14-77) (Table 1). One hundred forty-seven of 181 participants from the FDF were US based. Eleven participants were adolescents, with a median age of 17 years; 8/11 (72.7%) were female. Eighty-six (34.5%) of the subjects reported that they had monostotic FD, whereas 114 (45.8%) experienced polyostotic FD; the remaining 49 (19.7%) did not report their FD type.

**Table 1.** Demographic and disease characteristics of participants

	Combined cohort	FDF cohort	RUDY cohort
Total subjects, n	249	181	68
Women	205 (82.3)	154 (85)	51 (75)
Median age, years (range)^a^	40 (14-77)	38 (14-77)	48.5 (18-77)
Type of FD			
Monostotic	86 (34.5)	73 (40.3)	13 (19.1)
Polyostotic	114 (45.8)	97 (53.6)	17 (25.0)
No FD type available	49 (19.7)	11 (6.1)	38 (55.9)

Data are given as n (%) unless otherwise indicated.

Abbreviations: FD, familial dysautonomia; FDF, Familial Dysautonomia Foundation; RUDY, Rare and Undiagnosed Diseases study.

^a^Includes 11 adolescent patients, median age 17 years (range 14-17).

Data were missing for HADS in 17 individuals from FDF and 6 individuals from RUDY, and data were missing for PSQI in all individuals from FDF.

### Frequency and Type of Pain

**Figure 1.**
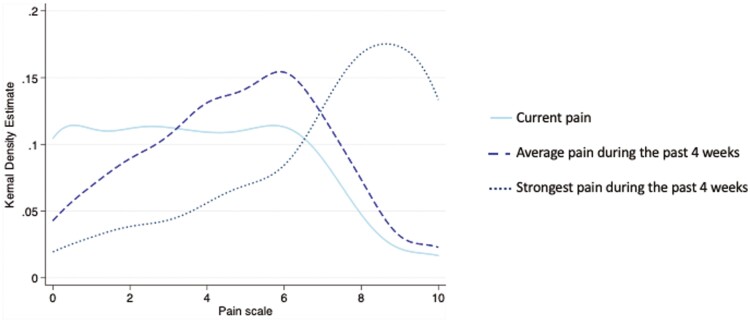
Distribution of current, average and strongest pain severity in 249 participants with fibrous dysplasia/McCune-Albright syndrome. Each line describes the smoothed probability estimate of the frequency distribution for reported current, average, and strongest pain during the last past 4 weeks from the painDETECT questionnaire.

The distribution of current pain severity and average and strongest pain during the previous 4 weeks are shown in Figure 1. While the distribution for current and average pain was normally distributed, strongest pain had a more bimodal distribution. Women reported higher current (3.9, *P* = 0.08), average (4.9, *P* = 0.02) and strongest (7.1, *P* = 0.07) pain experienced in comparison to the men (Table 2). Age was not associated with strongest (*P* = 0.76), average (*P* = 0.92), or current pain (*P* = 0.19) as measured by PD-Q.

**Table 2.** painDETECT scores of participants

	Current pain	Average pain in last 4 weeks	Strongest pain in last 4 weeks	Nociceptive pain	Unclear pain	Neuropathic-like pain
All	3.7 ± 2.7	4.7 ± 2.5	7.0 ± 2.7	113 (45.4)	58 (23.3)	78 (31.3)
Sex						
Men	3.2 ± 2.9	4.0 ± 2.7	6.3 ± 3.0	25 (56.8)	12 (27.3)	7 (15.9)
Women	3.9 ± 2.6	4.9 ± 2.4	7.1 ± 2.6	88 (42.9)	46 (22.4)	71 (34.6)
* P*-value for sex difference	0.09	0.02	0.07	0.05	0.05	0.05
FD type						
Monostotic	3.2 ± 2.6	4.1 ± 2.5	6.4 ± 3.0	41 (47.7)	20 (23.3)	25 (29.1)
Polyostotic	4.1 ± 2.7	5.1 ± 2.2	7.4 ± 2.4	47 (41.2)	29 (25.4)	38 (33.3)
No FD type available	3.9 ± 2.8	5.0 ± 2.7	7.0 ± 2.7	25 (51.0)	9 1(8.4)	25 (51.0)
* P*-value for monostotic vs polyostotic	0.02	0.003	0.04	0.67		

Data are expressed as mean ± SD or n (%) unless otherwise indicated. Nociceptive, unclear, and neuropathic-like pain defined by painDETECT questionnaire scores ≤ 12, 13-18, and ≥19 (33). Chi-squared used to test for significance.

**Figure 2.**
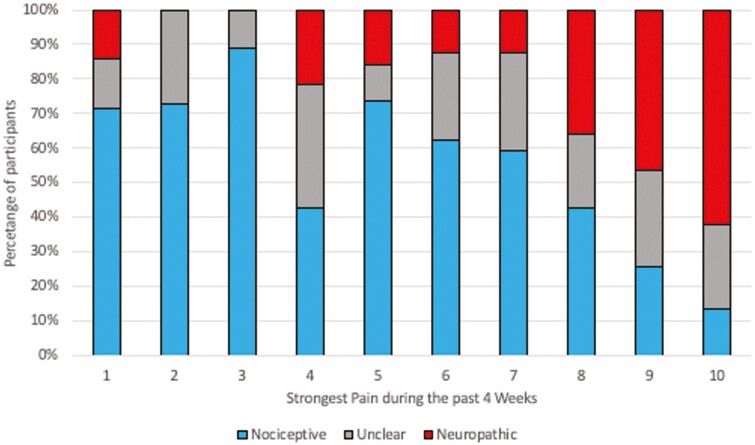
Proportions of nociceptive, unclear, and neuropathic-like pain for each severity of strongest pain strength during the past 4 weeks. Within each level of strongest pain during the past 4 weeks from 1 to 10, the proportion of individuals within in each pain with nociceptive, unclear, or neuropathic is shown as defined by the painDETECT questionnaire (33)

Based on the PD-Q, 113 (45.4%) participants satisfied the criteria for nociceptive pain, 78 (31.3%) satisfied criteria for neuropathic pain, and the remaining 58 (23.2%) respondents were categorized as unclear pain. The proportion of participants with neuropathic-like pain increased with higher mean of current, average, and strongest pain in the previous 4 weeks (*P* < 0.01), such that 64% of the total cohort with an average pain ≥8/10 had neuropathic-like pain (Figure 2). The prevalence of neuropathic pain was similar in adolescent (27.3%) vs adults (31.5%), although number of adolescents was small (*P* = 0.86). There was no difference in pain types between registries (*P* = 0.26).

The proportion of nociceptive, unclear, and neuropathic differed by sex. Women were less likely to score within the nociceptive range (42.9% women vs 56.8% men, *P* = 0.05) and more likely to report neuropathic-like pain (34.6% women vs 15.9% men, *P* = 0.05). The subset of subjects with polyostotic FD also reported more severe current, average, and strongest pain compared to the subset of subjects with monostotic FD (Table 2). However, there was no difference in categorization of pain as nociceptive, neuropathic, or unclear between participants with monostotic FD and the subset of subjects with polyostotic disease (*P* = 0.67).

### Association of Neuropathic-like Pain on Other Reported Measures of Quality of Life

Those with neuropathic-like pain reported significantly lower quality of life across all dimensions of the Medical Outcomes Study 36-Item Short Form Health Survey (Table 3). Moderate or severe anxiety, as measured by the HADS, was present in 34% of participants overall and in 42% of those with neuropathic pain (Table 3). Seventy-five percent of participants reported impaired sleep, as measured by the PSQI, with significantly (*P* = 0.001) worse score in those with neuropathic pain. In multivariate models, neuropathic pain remained an independent predictor of moderate/severe anxiety and depression but not impaired sleep (Table 4).

**Table 3.** Differences in other measures of participant-reported outcome measures

	Overall	Nociceptive	Unclear	Neuropathic	*P*-value
HAD anxiety score (n = 230)	8.4 ± 4.7	6.5 ± 4.2	8.8 ± 4.6	10.9 ± 4.5	<0.0001
Severe anxiety	26 (11.3)	3 (2.9)	5 (9.1)	18 (25.4)	<0.0001
HAD depression score^a^(n = 226)	6.2 ± 4.7	4.1 ± 3.8	6.5 ± 4.7	9.0 ± 4.4	<0.0001
Severe depression	13 (5.8)	1 (1.0)	5 (9.2)	7 (9.8)	0.0065
SF-36 physical function	60 (30, 85)	70 (55, 90)	60 (30, 85)	35 (15, 60)	0.0001
SF-36 role limitation due to physical health	25 (0, 75)	75 (0, 100)	0 (0, 50)	0 (0, 25)	0.0001
SF-36 role limitation due to emotional health	66.7 (0,100)	100 (33.3, 100)	50 (0,100)	0 (0, 66.7)	0.0001
SF-36 energy/fatigue	35 (15, 55)	50 (25, 65)	35 (15, 45)	17.5 (10, 35)	0.0001
SF-36 Emotional well-being	64 (44, 80)	75 (60, 88)	64 (44, 80)	48 (36, 68)	0.0001
SF-36 Social Functioning^b^	62.5 (31.3, 93.8)	87.5 (50,100)	50 (37.5, 75)	31.3 (6.3, 50)	0.0004
SF-36 Pain	45 (22.5, 67.5)	65 (45, 77.5)	45 (22.5, 57.5)	22.5 (17.5, 43.8)	0.0001
SF-36 General Health	40 (25, 65)	55 (35, 75)	40 (30, 55)	20 (10, 40)	0.0001
PSQI^b^ (n = 68)	9.7 ± 3.9	7.9 ± 3.4	11.4 ± 3.5	11.9 ± 3.8	0.0019
Sleep impairment^b^	51 (75.0)	21 (60.0)	16 (94.1)	14 (87.5)	0.039
PD-Q current pain (n = 249)	3.7 ± 2.7	2.4 ± 2.2	4.0 ± 2.3	5.5 ± 2.6	<0.0001
PD-Q strongest pain (n = 249)	7.0 ± 2.7	5.8 ± 2.8	7.3 ± 2.4	8.5 ± 1.8	<0.0001
PD-Q average pain (n = 249)	4.7 ± 2.5	3.5 ± 2.3	5.0 ± 2.0	6.3 ± 2.0	<0.0001

Data are given as n (%), median (interquartile range), or mean ± SD. Nociceptive, unclear, and neuropathic-like pain defined by PD-Q scores ≤12, 13-18, and ≥19, respectively (33). Severe anxiety or depression are defined by scores of 15-21; sleep impairment, by score >8. *P*-values represent difference between the PD-Q groups using parametric and nonparametric tests.

Abbreviations: FDF, Familial Dysautonomia Foundation; HADS, Hospital Anxiety and Depression Scale; PD-Q, painDETECT questionnaire; PSQI, Pittsburgh Sleep Quality Index; RUDY, Rare and Undiagnosed Diseases study; SF-36, Short Form 36.

^a^Data was missing for HADS in 17 individuals from FDF and 6 individuals from RUDY.

^b^Data for PSQI and SF-36 social functioning was only available from RUDY participants.

**Table 4.** Type of pain and depression, anxiety, and sleep impairment

	Adjusted for age and sex		Adjusted for age, sex, and strongest pain during last 4 weeks	
	Unclear	Neuropathic	Unclear	Neuropathic
Moderate/severe anxiety (n = 230)	3.4 (1.6-7.5)	6.0 (2.9-12.3)	3.3 (1.5-7.4)	5.6 (2.5-12.3)
Moderate/severe depression (n = 230)	2.4 (0.9-6.6)	6.9 (2.8-17.2)	1.6 (0.5-4.5)	3.7 (1.4-10.0)
Sleep impairment (n = 68)^a^	12.4 (1.4-108.1)	4.7 (0.9-25.4)	10.6 (1.2-93.3)	3.6 (0.63-21.1)

Odds ratios (95% CIs) are shown with 2 models: adjusted for age and sex and then additionally adjusted for strongest pain during the past 4 weeks. Nociceptive, unclear, and neuropathic-like pain defined by PD-Q scores ≤12, 13-18, and ≥19 respectively (33). Nociceptive pain used as the referent group.

Abbreviation: RUDY, Rare and Undiagnosed Diseases study.

^a^This model is limited to only the RUDY cohort (n = 68), which represents 27% of the whole cohort.

## Discussion

We report for the first time, a high prevalence of neuropathic-like pain in patients with FD/MAS. Neuropathic-like pain was more common in participants with higher pain severity, and the presence of neuropathic-like pain correlated with anxiety, depression, quality of life, and impaired sleep, although the latter and SF-36 social functioning was only recorded in the RUDY study. This represents an important potential unmet need for treatment. These findings suggesting a neuropathic-like component to pain may partially explain previous clinical observations in FD demonstrating that pain does not correlate with the burden of skeletal disease and is more frequent and severe in adults than in children (4, 9).

Pain is a key symptom of FD, and yet there is little evidence to support its management. Currently most research is focused on the use of bone-specific therapies such as antiresorptives (6), with average pain severity as the key clinical endpoint. However, bone pain is a complex subjective experience with increasing evidence for contributions from abnormal nerve sprouting, spinal, and central processes in addition to peripheral nociception (5). The only randomized trial of high-dose oral bisphosphonates in FD/MAS, which was negative, recruited patients on the severity of pain and not its character (6). A neuropathic component to FD pain could potentially explain the lack of efficacy in this study.

The clinical consequences of underappreciation of chronic and neuropathic-like pain are significant (28). Neuropathic-like pain is a common and variable complication of many disease processes. However, the prevalence of neuropathic-like pain noted in this study (31.3%) was high in comparison to studies in rheumatoid arthritis (5%) and osteoarthritis (6.7%) (29-32) but slightly less than those in low back pain (37%) (33). Patients with neuropathic-like pain may benefit from agents targeting the nervous system, such as tricyclic antidepressants, serotonin-noradrenaline reuptake inhibitors, pregabalin, and gabapentin (34), in addition to the antiresorptive agents and analgesics (2, 35-37) that currently form the mainstay of treatment (38). In this study patients with neuropathic-like pain had worse quality of life, mental health, and sleep quality in comparison to those with nociceptive pain even after adjusting for the severity of the pain. Particularly striking is the finding that 25% of those in the neuropathic-like pain group had severe anxiety, and nearly 1 in 10 had severe depression, higher than rates in the general population (39). This is supported by previous studies of neuropathic-like pain where anxiety and depression are common (40). However, in keeping with a previous study in patients with FD (7), overall mean scores for anxiety and depression are low and fall either within normal or mild anxiety/depression. This is in contrast to other patients with chronic pain, where high proportions have severe anxiety or depressive disorders (41). This suggests a dichotomy where many patients with FD do not experience adverse mental health outcomes, but those with neuropathic-like pain form a group where adverse mental health is more likely. Awareness of this among those with a neuropathic component to their pain would allow better screening and treatment of this group.

Sleep problems are common in the community (42) and are associated with mental (depression, anxiety) and physical (cardiovascular disease, stroke, obesity, hypertension) comorbidities as well as increased mortality (43, 44). The average PSQI scores reported here, in those with and without neuropathic-like pain, are comparable with the scores reported for patients with chronic back pain referred to a tertiary rehabilitation center (45) and worse than reported for patients undergoing bone marrow transplantation or with breast cancer (24). Our findings highlight the need to include assessment and treatment of sleep problems in adults with FD/MAS.

Previous studies have examined average pain using the Brief Pain Inventory (4). One advantage of the PD-Q is that it also includes numerical rating scales asking participants to rate their current pain, average over the previous 4 weeks, and at its strongest over the last 4 weeks. This is the first study to assess aspects of pain in patients with FD/MAS. Given the distributions, strongest pain during the last 4 weeks better identifies a subgroup with more severe pain. Further work is needed to understand which measure patients would prioritize and would inform inclusion criteria and outcomes for clinical studies of pain in FD/MAS.

Compared to subjects with monostotic FD, those with polyostotic FD reported that they experienced more severe average and strongest pain, despite having comparable rates of nociceptive and neuropathic-like pain. A previous study has suggested that pain severity does not necessarily correlate with FD disease burden. However, we did not have radionuclide bone scan data to provide objective measures for the extent of skeletal disease.

The mechanisms of bone pain in FD/MAS are unknown. Osteoclasts are known to predominate in FD lesions from increased levels of receptor activator of nuclear factor kappa-B ligand (46, 47). It is thought that the osteoclasts’ creation of an acidic environment, in excess, triggers the local transient receptor potential channel vanilloid subfamily member 1 and acid-sensing ion channels and thus the perception of pain (48). This may contribute to the pain-relieving properties of bisphosphonates and denosumab in FD/MAS and other conditions with excessive osteoclast activity (5, 49-51). Physiologically, there is rationale to support a neuropathic component to pain in FD (5). Bone appears to have a distinct pattern of sensory innervation, consisting of thinly myelinated A-delta and unmyelinated CGRP + nerve fibers that highly express Trk A, a receptor for the ligand nerve growth factor (NGF) (5). Work in models of cancer bone pain has demonstrated NGF-dependent ectopic sprouting of nerve fibers contributes to bone pain (5, 52). Such nerve fiber sprouting can be found as a normal response to bone trauma, where it likely plays a protective role (49). However, it has also been demonstrated in the chronic pain states of osteoarthritis and intervertebral disc pain, perhaps as a result of failure to prune back such fibers when no longer required (49, 53, 54). Whether such ectopic nerve fiber sprouting could contribute to neuropathic bone pain in FD is unclear. In addition to promoting ectopic nerve fiber sprouting, NGF promotes bone pain through sensitization of sensory nerve fibers, and anti-NGF treatments have shown efficacy in reducing pain from osteoarthritis and low back pain (49, 55-57). In addition, central sensitization has not been studied in FD, but its role in other chronic pain states makes it a likely contributing factor to neuropathic and chronic pain in FD.

Another potential mechanism involves IL-6, which has been shown to affect receptor activator of nuclear factor kappa-B ligand expression and osteoclastogenesis (58, 59). IL-6 has also been shown to be present at elevated levels in response to the *GNAS* mutation (11, 60). IL-6 is thought to contribute to the generation of bone cancer pain, through overexcitability of dorsal root ganglion neurons and upregulation of transient receptor potential channel vanilloid subfamily member 1 in dorsal root ganglion neurons (10). In the setting of peripheral nerve injury, IL-6 has been shown to contribute to nerve ending sprouting (10). Importantly, anti-IL-6 treatments are already in common clinical usage for the treatment of rheumatoid arthritis and have shown promising pain relief in a case report in FD; a clinical trial is ongoing (ClinicalTrials.gov identifier: NCT01791842).

A major strength of our study is the high participant numbers in this rare disease. This is the largest study of pain in FD/MAS to date, and these novels findings have the potential to immediately and directly impact patient care. Results were replicable across 2 large international cohorts. This study also included the participation of a small group of adolescents, a frequently underrepresented group, whose responses were comparable to a purely adult cohort. However, we acknowledge several limitations. The registries did not have a central identification system or country of origin, so it is possible that there may be overlap of patients between the RUDY and FDF registries. The diagnosis of FD/MAS was patient-reported without additional confirmatory clinical information. Given the use of patient reported data, it is possible that patients with more severe disease, including pain or mental health presentations, contribute more to registry data. This potentially explains the high prevalence of polyostotic FD in this cohort (45.8%), indicating the data are likely biased toward those with a higher disease burden. Additional medical diagnoses other than FD were not considered in the evaluation of the neuropathic pain, and lack of clinical data prevented more detailed assessment of the pain type, skeletal sites affected, and use of analgesic medications that may have affected pain (61). We recognize inclusion of higher disease burden patients could lead to an overestimation of the prevalence of neuropathic-like pain while the use of specific analgesics could lead to an underestimation. We did not have data on complications of FD that can potentially contribute to the development of pain, such as fractures, postsurgical changes, and hypophosphatemia (62) (63), and further studies are needed to determine the role of these complications in the development of overall and neuropathic-like pain in FD. While the PD-Q has been validated for musculoskeletal disorders such as posttraumatic/postsurgical neuropathy, it has not yet been specifically validated for FD/MAS (64), and future research should validate this tool for neuropathic pain in individuals with FD/MAS. Another limitation is that the PD-Q is restricted to the 4 weeks prior to assessment and therefore may underestimate the effects of less frequent superimposed acute flares of pain. The study was limited by the use of the PD-Q without confirmatory neurological examination and testing, hence our use of the term “neuropathic-like pain.” We acknowledge that the features of neuropathic pain identified using the PD-Q may actually reflect nociplastic pain, a term introduced by the international pain community of pain researchers as a mechanistic descriptor for chronic pain states that are not typically nociceptive or neuropathic in origin but where clinical and psychophysical findings are consistent with altered nociceptive function (65, 66). Currently, we lack knowledge regarding potential FD-induced damage of the somatosensory nervous system, and mechanistic studies are warranted to establish whether the neuropathic-like pain reported by the FD patients reflects nerve damage. Further, we were unable to exclude mixed patterns of pain including nociplastic pain (67, 68), and further confirmatory research is required. There were also some inconsistencies in data collection: the PSQI was only available for RUDY participants, and there was missing data for the HADS responses. Finally, these data are cross-sectional and therefore can only highlight associations, not the direction, of effect and are therefore hypothesis generating.

Our findings demonstrate that neuropathic-like pain is common in patients with FD/MAS and is associated with worse quality of life, increased rates of severe anxiety and depression, and poor sleep quality, as based on the RUDY participants’ data. We suggest that patients with FD/MAS should be evaluated for neuropathic-like pain, mental well-being, and sleep disturbance; may benefit from treatments targeting neuropathic pain; and should be included within eligibility or baseline stratification for clinical trials.

## Data Availability

Anonymized data from the Rare and Undiagnosed Diseases study is available by direct request using www.rudystudy.org site and accessing the researcher tab. Data from the Fibrous Dysplasia Foundation requires a data request from the Fibrous Dysplasia Foundation.
